# Special Issue “Molecular Research in Rice, 2nd Edition”

**DOI:** 10.3390/ijms27010078

**Published:** 2025-12-21

**Authors:** Prasanta K. Subudhi

**Affiliations:** School of Plant, Environmental and Soil Sciences, Louisiana State University, Baton Rouge, LA 70803, USA; psubudhi@agcenter.lsu.edu; Tel.: +1-2255781303

## 1. Introduction

Rice (*Oryza sativa* L.) is recognized as the most crucial food crop worldwide, playing a central role in global food security as the staple diet for over half of humanity, as well as holding profound cultural, economic, and nutritional importance [1]. Sustaining rice productivity and quality has become increasingly challenging due to climatic disturbances, evolving consumer preferences, and rising biotic and abiotic stresses. Consequently, a multi-disciplinary and multi-dimensional research approach is essential for developing rice varieties suited to the future. Rapid advances in molecular tools and omics technologies have been vital in dissecting the genetic basis of complex agronomic traits and designing effective breeding strategies for developing novel varieties with high yield, superior grain quality, enhanced nutritional attributes, and improved climate resilience.

Plant breeding is a continuous process that requires innovative strategies and tools to accelerate the development of new varieties, with progress across multiple fields being essential in achieving this goal. Rice researchers are increasingly integrating biotic and abiotic stress assessment, genetics, agronomic trait improvement, and multi-omics approaches such as metabolomics and transcriptomics to enhance resilience, quality, and productivity [2]. Such multi-omics strategies provide a comprehensive perspective on rice physiology, linking gene expression patterns with metabolic pathways that govern complex agronomic traits. Integrative studies have revealed key regulatory networks and candidate genes that can be targeted in breeding programs, offering new strategies for sustainable rice production under diverse environmental conditions. Both marker-assisted selection and genome editing, particularly CRISPR-based approaches, are emerging as powerful tools for translating genetic insights into actionable targets for rice improvement [3,4].

This Special Issue of the *International Journal of Molecular Sciences*, “Molecular Research in Rice, 2nd Edition”, brings together seven original research articles and one review. Collectively, these contributions highlight the application of marker-assisted selection, omics tools, and genome editing in untangling the genetics of grain quality, nutritional quality, biotic and abiotic stress tolerance, tillering, and nitrogen-use efficiency.

## 2. Leveraging Marker-Assisted Pyramiding for Durable Disease Resistance

Marker-assisted selection (MAS) has been increasingly employed to pyramid multiple agronomically important traits controlled by major genes [5]. A central focus of contemporary rice research is the improvement of biotic stress resistance in elite cultivars. Although Pusa Basmati 1509 (PB1509) is a leading Basmati variety in India, contributing substantially to foreign exchange earnings due to its exceptional cooking quality, aroma, and early maturity, it continues to exhibit high vulnerability to bacterial blight (BB) and blast diseases. In their study, Singh et al. (contribution 1) introgressed multiple bacterial blight resistance genes (*xa13*, *Xa21*, *Xa38*), along with blast resistance genes (*Pi9*, *Pib*, *Pita*), into the genetic background of PB1509 via marker-assisted backcross breeding, resulting in near-isogenic lines with high genomic similarity and stable agronomic performance. Foreground selection was conducted using gene-linked markers, and recurrent parental background recovery in advanced generations was assessed using the 80K SNP Rice Pan Genome Array. Pyramiding of multiple resistance genes is expected to enhance the durability of this popular Basmati variety by slowing pathogen evolution through broad-spectrum resistance. This study underscores the potential of molecular breeding to strengthen resistance against multiple diseases in widely adapted Basmati rice varieties, thereby reducing chemical inputs and promoting economic stability, ecological sustainability, and global competitiveness.

## 3. Studies Deciphering Molecular Basis of Grain Quality, Aroma, and Nutritional Characteristics

Fragrance is a highly valued trait in rice, directly influencing consumer preference and accounting for approximately 20% of global rice trade. Rice varieties such as Jasmine and Basmati are prized for their fragrance and higher prices, making aroma a key focus for breeders. The compound 2-acetyl-1-pyrroline (2AP) is the predominant contributor to rice fragrance [6], and its biosynthesis involves multiple precursors (proline, glutamic acid, ornithine, 1-pyrroline) and enzymes (P5CS, GAPDH), underscoring its complexity as a multifaceted metabolic process. Mutations in the *BADH2* gene are strongly associated with fragrance, as null alleles prevent the conversion of precursors into GABA, leading to the accumulation of intermediates that enhance 2AP production [7]. Despite significant progress, the network of genes and metabolites regulating fragrance remains incompletely understood, particularly in diverse landraces with rich genetic variation. Zeng et al. (contribution 2) integrated metabolomic and transcriptomic analyses across four developmental stages (seedling, reproductive, filling, maturation) to unravel the molecular genetic basis of fragrance in the Guangxi landrace Shangsixiangnuo (SSXN). Their study confirmed the 806 bp deletion in *BADH2* as the genetic basis of SSXN’s fragrance, identified stage-specific metabolites and differentially expressed genes (DEGs), and highlighted 2AP-associated regulatory networks. Notably, the downregulation of *P4H4* (procollagen-proline dioxygenase) suggests a role in proline metabolism and its connection to 2AP biosynthesis. This work underscores the value of landraces such as SSXN as reservoirs of genetic diversity and unique traits, while providing new molecular targets and metabolite markers for breeding programs aimed at enhancing rice aroma quality and consumer appeal.

Chalkiness is a major quality trait that reduces grain transparency, thereby negatively affecting appearance, cooking quality, and nutritional value, ultimately diminishing both market value and consumer acceptance. *OsFLO2* was previously identified as a gene influencing chalkiness [8]; however, its regulatory mechanism has not yet been fully elucidated. Tang et al. (contribution 3) identified a bHLH transcription factor, *OsFIF3*, as a key regulator and provided clear evidence of its interaction with *OsFLO2* in controlling starch metabolism and rice chalkiness. *OsFIF3* overexpression resulted in increased chalkiness, hollow starch granules, and reduced grain weight due to defective starch filling, directly linking it to grain quality deterioration. Their study further demonstrated that *OsFIF3* binds to the CACGTG motif on the promoters of key genes (*FLO2*, *SUT1*), suppressing their expression and thereby disrupting starch metabolism and energy allocation. Additionally, *OsFIF3* is conserved in monocots but variable in dicots, suggesting a specialized role in rice and related cereals, and nuclear localization confirmed its function as a transcriptional regulator. The significance of this study lies in its uncovering of a novel molecular mechanism underlying rice chalkiness, providing valuable insights for breeding high-quality rice varieties with improved grain appearance, cooking quality, and yield stability.

Given the dominance of rice in the daily diet of much of the world’s population, there is increasing emphasis on improving its nutritional quality. Prolamins, which account for 20–30% of seed storage proteins, are considered indigestible [9] and have been associated with allergic reactions similar to those caused by wheat, barley, and rye proteins. They are also nutritionally inferior to glutelins due to deficiencies in essential amino acids such as lysine and methionine. Reducing prolamin content is therefore critical for enhancing rice digestibility, nutritional value, and consumer safety. Seed storage protein composition has additionally been implicated in seed development and germination. Notably, mutant rice lines with reduced prolamin levels have demonstrated higher efficiency in recombinant protein production, underscoring their potential utility in biotechnology and therapeutic protein applications. Although RNAi and RNA-silencing approaches have been employed to lower prolamin levels, the regulatory mechanisms governing specific 13 kDa prolamin subgroups during seed development and stress responses remain poorly understood. Pham et al. (contribution 4) applied CRISPR-Cas9 for the first time to selectively edit 13 kDa prolamin genes in rice, thereby providing novel insights into seed biology and protein storage mechanisms essential for crop improvement. By knocking out the *Pro13a-I* and *Pro13b-I*/*II* subgroups of 13 kDa prolamins, the authors developed four knockout rice lines. These mutants showed higher levels of glutelins and other prolamins, indicating regulatory interactions among seed storage proteins (SSPs). Mutant lines exhibited reduced grain weight, altered starch composition, and atypical starch granules and protein bodies, implicating prolamins in seed morphology and storage structure. Transcriptome analysis revealed 746 differentially expressed genes, including ER-stress-related genes (*BiP*, *PDI*, *CNX*) and transcription factors (*RPBF*, *RISBZ1*, *OsNAC20*/*26*), while negative correlations between *Pro13a-I* and other prolamin subgroups further suggest functional antagonism in their regulation. This study uncovered the role of prolamins in seed development, protein storage, and stress regulation. The findings highlight compensatory protein expression, altered starch composition, and ER stress responses, offering deep mechanistic insights into prolamin function and opening new pathways for the development of nutritionally superior rice varieties.

## 4. Molecular Genetics of Tillering Response to High-Temperature Stress and Variable Nitrogen Fertilization

The number of productive panicles is a critical determinant of rice yield. High-temperature stress during the tillering stage impairs tiller bud growth, leading to the formation of weak panicles. Early tillering vigor (ETV) traits are therefore essential for enabling tiller buds to withstand such stress and develop into effective panicles, thereby contributing to increased grain yield. Although the molecular mechanisms underlying tiller bud formation have been extensively studied [10], the genetic regulation of ETV traits under stress conditions remains largely unexplored. Using QTL mapping and bulk-segregant analysis by resequencing (BSA-seq) in conjunction with a backcross inbred line (BIL) population, Wu et al. (contribution 5) identified multiple QTLs associated with ETV across chromosomes 1, 2, 3, 4, 5, 7, and 9. An overlapping QTL interval spanning 1.39 Mb on chromosome 4 was pinpointed as a key region regulating ETV traits. Six genes (*Os04g0455650*, *Os04g0470901*, *Os04g0500600*, *Os04g0456900*, *Os04g0506800*, and *Os04g0497300*) were found to be differentially expressed between ETV and late tillering vigor (LTV) lines, and missense mutations detected in parental genomic DNA further suggest that these genes are strong candidates for regulating ETV. The valuable genomic resources generated in this study will be useful for the application of marker-assisted selection (MAS) to improve rice resilience under high-temperature stress.

Nitrogen fertilizers are indispensable for sustaining rice productivity; however, excessive application reduces efficiency, causes environmental damage, and lowers grain yield due to resource competition among tillers [11,12]. Developing rice varieties that achieve high yield with reduced nitrogen input has therefore become a major breeding objective in the context of shrinking arable land and the urgent need to ensure food security for a growing global population. The number of effective tillers directly dictates panicle production per plant, exerting a major influence on yield potential. To dissect the genetic basis of this complex quantitative trait, Liu et al. (contribution 6) conducted a genome-wide association study (GWAS) using 469 diverse germplasm accessions, encompassing both *japonica* and *indica* rice, and identified QTLs associated with effective tillering under both low- and high-nitrogen conditions. Seven candidate genes (*NAL1*, *OsCKX9*, *Os01g0690800*, *Os02g0550300*, *Os02g0550700*, *Os04g0615700*, *Os04g06163000*) were identified as strong regulators of nitrogen-responsive tillering, and the integration of haplotype analysis with spatio-temporal expression profiling refined candidate gene identification, thereby strengthening confidence in their functional roles. QTL validation across multiple years and treatments further ensured robustness. This enhanced understanding of the genetic architecture underlying nitrogen response in rice tillering provides valuable genomic resources for marker-assisted selection (MAS) aimed at improving yield and nitrogen-use efficiency, directly addressing the global challenge of producing more rice with reduced reliance on chemical fertilizers.

## 5. Key Flowering Gene *Hd3a* Promotes Tiller Formation

Since flowering influences both panicle number and size, Zheng et al. (contribution 7) investigated the role of the key flowering gene *Hd3a*, a central regulator of reproductive timing and yield potential, in tiller bud formation. While *Hd3a*’s role in floral transition is well established [13], its mechanism in promoting branching and tiller bud outgrowth has remained unclear. These authors uncovered a novel molecular mechanism by which *Hd3a* promotes rice tiller bud outgrowth, finding that overexpression of *Hd3a* accelerated flowering and stimulated tiller bud outgrowth, whereas knockout delayed both processes. Using BioID proximity labeling, *Hd3a* was shown to interact with D14, D53, and TPR proteins, which are core components of the strigolactone (SL) signaling pathway, attenuating the inhibitory effect of strigolactones on tillering by preventing D53 degradation induced by the SL analog rac-GR24. Expression of SL biosynthesis and signaling genes (*OsD27*, *OsD3*, *OsD14*, *OsD53*) was significantly altered in *Hd3a*-overexpressing (Hd3aOE) lines and *hd3a* mutants, while the downstream target *OsFC1* (*OsTB1*), a negative regulator of tillering, was suppressed in Hd3aOE plants and upregulated in *hd3a* mutants, confirming *Hd3a*’s role in modulating SL signaling. Collectively, these findings suggest new genetic targets for breeding rice varieties with improved plant architecture and higher yield by linking floral transition with branching regulation.

## 6. Role of Glutaredoxin in Rice Growth, Development, and Stress Resistance

Reactive oxygen species (ROS) function as crucial signaling molecules for development and stress responses, yet they also act as toxic byproducts when present in excess [14]. Both biotic and abiotic stresses trigger ROS accumulation, which can damage macromolecules and disrupt signaling pathways, and with climate change intensifying, effective management of ROS homeostasis has become essential to sustaining crop productivity. Rice has evolved multiple antioxidant defense mechanisms, among which the glutaredoxin (GRX) system is particularly important for regulating protein function and maintaining redox balance. A review article by Zhai et al. (contribution 8) in this Special Issue consolidated current knowledge, emphasizing the critical role of GRXs in enhancing rice resilience against diverse stressors while also regulating key developmental processes. The rice genome contains 48 GRX gene loci, and classification of these loci into four categories (CPYC, CGFS, CC, and GRL), based on their active sites, underscores their structural and functional diversity. Recent studies further demonstrate GRX involvement in redox regulation, endoplasmic reticulum (ER) stress responses, and protein folding. Collectively, these insights provide a roadmap for future genetic improvement programs, targeting GRX-mediated pathways to develop superior rice varieties capable of withstanding climate-induced stresses.

## 7. Conclusions and Future Directions

The above studies have highlighted the benefits of integrating marker-assisted breeding, CRISPR-Cas9 mediated genome editing, QTL mapping, GWAS, and multi-omics approaches, which collectively provide genetic and biochemical insights into diverse attributes, including disease resistance, abiotic stress tolerance, grain chalkiness, nutritional quality, and tillering. In the process, valuable genomic resources have been generated to advance both basic and translational research in rice. These findings deepen our understanding of the genetic architecture underlying traits that are critical to consumer preference, yield improvement, and climate resilience. Furthermore, molecular targets have been identified for optimizing tiller dynamics, enhancing yield stability under diverse agronomic conditions, and improving grain quality and nutritional characteristics.

The collection of articles in this Special Issue delineates several prospective research avenues to pursue for future rice improvement. Introgression of additional disease- and pest-resistance genes through MAS will enhance durability of rice cultivars. To reduce chalkiness in rice grains, efforts should focus on identifying favorable haplotypes, employing gene editing to downregulate *OsFIF3*, and investigating its interaction with other transcriptional networks regulating starch biosynthesis. Given that several candidate genes and metabolites associated with early tillering vigor, nitrogen-use efficiency, aroma, and chalkiness have been identified, these candidates should first be functionally validated before being deployed in marker-assisted breeding. Moreover, the impact of these genes under diverse environmental conditions should be investigated to optimize their expression. CRISPR/Cas-based strategies can be leveraged to fine-tune key genes (BADH2, prolamins, GRX) and their related pathways to enhance fragrance, nutritional quality, and climate resilience without compromising yield. Similarly, prolamin editing combined with fortification of essential amino acids represents a promising strategy to enhance rice nutritional quality.

## Data Availability

Not applicable.
